# *Dictyostelium* AMPKα regulates aggregate size and cell-type patterning

**DOI:** 10.1098/rsob.170055

**Published:** 2017-07-12

**Authors:** Ranjana Maurya, Rakesh Kumar, Shweta Saran

**Affiliations:** School of Life Sciences, Jawaharlal Nehru University, New Delhi 110067, India

**Keywords:** AMPKα, *Dictyostelium*, aggregate size, cell-type patterning

## Abstract

Starved *Dictyostelium* cells aggregate into groups of nearly 10^5^ cells. AMPK is a highly conserved serine/threonine protein kinase consisting of a catalytic and two regulatory subunits. As multi-cellular development in *Dictyostelium* is initiated upon starvation, we explored the role of the energy sensor, AMPK, which shows significant similarity to human AMPK and is expressed throughout development. Deletion of the *ampkα* gene results in the formation of numerous small-sized aggregates that develop asynchronously to form few fruiting bodies with small sori and long stalks. On the other hand, *ampkα^OE^* cells form fruiting bodies with small stalks and large sori when compared with wild-type, Ax2. A minimum of 5% *ampkα^−^* cells in a chimaera with Ax2 cells was sufficient to reduce the aggregate size. Also, the conditioned media collected from *ampkα^−^* cells triggered Ax2 cells to form smaller aggregates. The starved *ampkα^−^* cells showed low glucose levels and formed large aggregates when glucose was supplied exogenously. Interestingly, *ampkα^−^* cells exhibit abnormal cell-type patterning with increased prestalk region and a concomitant reduction of prespore region. In addition, there was a loss of distinct prestalk/prespore boundary in the slugs.

## 1. Background

AMPK (5′ adenosine monophosphate-activated protein kinase) is a heterotrimeric serine/threonine kinase, comprising a catalytic (α) and two regulatory (β and γ) subunits, and is conserved from yeast to humans. It is both a nutrient and an energy sensor that helps maintain energy homeostasis. It plays a role in the regulation of cellular as well as whole-body energy metabolism and also participates in the cell-cycle and membrane excitability regulation. AMPK coordinates control of cell growth and autophagy, acting as a metabolic checkpoint, and inhibits cellular growth via suppression of the mTORC1 (mammalian target of rapamycin complex 1) pathway. It acts as a central regulator of cell growth, cell proliferation and development [1–5]. When activated, AMPK promotes energy-producing catabolic pathways while inhibiting anabolic pathways, such as cell growth and proliferation. Besides its role as an energy sensor, it also plays a role in cell differentiation and development of organisms [6]. In *Caenorhabditis elegans*, AMPKα promotes cell survival and arrests germline development during nutrient stress [7].

In the case of *Dictyostelium discoideum*, the size of multi-cellular structures formed is well regulated. *Dictyostelium* amoeba divides mitotically when food is abundant, but undergoes multi-cellular development upon starvation. Vegetative cells secrete prestarvation factor (PSF) that helps monitor cell density relative to the amount of available nutrients [8]. High PSF induces the expression of genes required for aggregation. When the food supply is depleted, PSF production declines and another cell density sensing factor called conditioned medium factor (CMF) begins to accumulate. Once the starving cells reach high cell density, CMF accumulates and the cells initiate aggregation via cAMP signal relay [9] to aggregate into groups of approximately 10^5^ cells. Hohl & Raper [10] had earlier investigated several small-sized aggregate mutants and found them to be defective in either aggregation or cell number or mass sensing. It was observed that mutants defective in aggregation could be rescued by crowding of the cells, so that aggregation becomes unnecessary. The cell number sensor senses the number of cells present in a group, and if they are exceedingly high it breaks them into smaller groups. Earlier, Brock & Gomer [11] observed the *smlA* mutants that formed small-sized aggregates, due to the oversecretion of Countin A protein. The aggregates then form the migratory slugs where the anterior quarter region is composed of prestalk cells and the remaining posterior region of prespore cells. The ratio of the cell types remains constant regardless of the size of the multi-cellular structures formed. Prestalk cells are further divided into subtypes: pstA cells occupy the anterior 10% of the slug, pstAB cells occupy the core to the tip, pstO cells are found behind the pstA cells and anterior-like cells (ALCs) lie dispersed within the prespore region [12]. A number of genes play a role in cell-type proportioning and spatial patterning [13–16], thus there is a large selective pressure on the starved cells to form fruiting bodies for proper spore dispersal. Neither too long nor too short fruiting bodies are advantageous for the organism. A fruiting body is composed of two terminally differentiated cell types, namely the stalk (dead vacuolated) cells and the spore (viable) cells [17].

AMPK plays an important role in starvation responses and nutrient deprivation is necessary for the initiation of development in this organism. Earlier, Bokko *et al*. [18] investigated the role of AMPK in mitochondrial diseases and found overexpression to result in fruiting bodies with short, thick stalks and comparatively large sori, while antisense inhibition developed fewer and smaller fruiting bodies.

This study was undertaken to explore the functions of AMPKα during development of *D. discoideum*, which was found to be involved in regulating the aggregate size. The *ampkα^−^* cells formed small-sized aggregates, which developed asynchronously and the spores formed displayed reduced viability. The developmental defects shown by *ampkα^−^* cells were cell autonomous as chimaeras formed with only 5% *ampkα^−^* mixed with 95% Ax2 cells caused the aggregation streams to break up. The conditioned medium (CM) collected from *ampkα^−^* cells caused the Ax2 cells to form small-sized aggregates. The *ampkα^−^* cells showed low cytosolic glucose levels during starvation and the small-aggregate phenotype could be corrected to a certain extent when developed in the presence of exogenous glucose. In chimaeras with Ax2 cells, the *ampkα^−^* cells showed a propensity towards the prestalk region and had lower tendency to form spores. Importantly, our results showed AMPKα to play a regulatory role in the spatial cell-type patterning as mutation caused an increase and mis-localization of the prestalk cells and a decrease in the prespore cells, ultimately resulting in fruiting bodies with small sorus and long stalk.

## 2. Results

### 2.1. *ampkα* mRNA is expressed in prestalk/stalk cells

**Figure 1.**
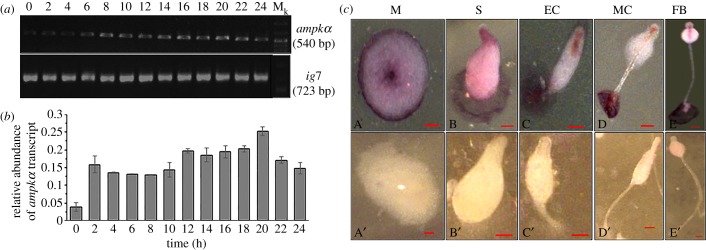
Spatio-temporal transcript patterns of *ampkα* during development. (*a*) Temporal expression pattern of *ampkα* and *rnlA* (*ig7*) by RT-PCR using cDNA samples at various time-points of development. (*b*) Relative abundance of *ampkα* to *rnlA* transcript at various time-points. (*c*) Spatial expression patterns as studied by *in situ* hybridization with *ampkα* antisense probe (A–E) and sense probe (A′–E′). M_k_, DNA marker; M, mound; S, slug; EC, early culminant; MC, mid-culminant; FB, fruiting body; scale bar, 50 µm; *n* = 3.

To determine the spatio-temporal mRNA expression patterns of *ampkα,* reverse transcriptase PCR (RT-PCR) and *in situ* hybridization analyses were performed. The *ampkα* transcript was present during growth and development, showing minimum levels in the vegetative cells and increased levels during multi-cellular development (figure 1*a,b*). It remained more or less constant during later stages of development. The whole mount *in situ* hybridization analyses showed *ampkα* transcript to be localized in the tip of the mound and at the site of contact with the substratum corresponding to the prestalk cells (figure 1*c*(A)). As development proceeded towards culmination, the transcript was localized in the pstAB cells, stalk tube and the basal disc region (figure 1*c*(B–E)). The high expression levels in the basal disc suggest it to be present largely in the pstB cells as they are shed off during culmination. The sense probe did not show any evident staining (figure 1*c*(A′–E′)). In conclusion, *ampkα* transcript shows prestalk localization and is expressed throughout growth and development.

### 2.2. Successful creation of *ampkα* mutants

To investigate the functions of AMPKα, we successfully made both overexpressing and deletion mutants (electronic supplementary material, figures S1 and S2). The constitutive promoter, *actin 15*, was used to drive the expression of the fusion protein AMPKα–Eyfp and was called AMPKα overexpressor (*ampkα^OE^*). Following transformation and blasticidin selection, 500 independent clones were isolated and screened for positional integration by PCR amplification of the genomic DNA. Four positive clones of *ampkα* null were obtained having similar growth and development patterns. One of these clones, designated as *ampkα^−^*, was used for further studies. Rescue (*ampkα^Res^*) was created by expressing the AMPKα fusion protein in *ampkα^−^* cells. The growth and early developmental defects shown by *ampkα^−^* were more or less rescued in *ampkα^Res^* cells.

### 2.3. AMPKα suppresses cell proliferation and growth

**Figure 2.**
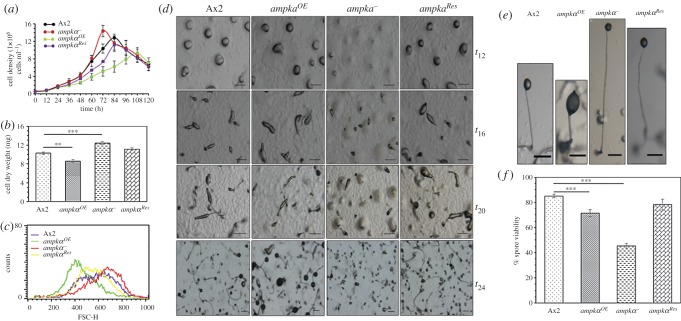
Overexpression and/or disruption of *ampkα* affect growth, development and spore viability. (*a*) Cell proliferation studies with *ampkα^OE^*, *ampkα^−^* and *ampkα^Res^* and their comparison with Ax2 cells (seeding density approx. 5 × 10^5^ cells ml^−1^). (*b*) Dry weight of 5 × 10^7^ cells harvested from Ax2, *ampkα^OE^*, *ampkα^−^* and *ampkα^Res^*. (*c*) Cell-size analysis of cells from various strains using FACS. (*d*) Time-specific developmental stages of Ax2, *ampkα^OE^*, *ampkα^−^* and *ampkα^Res^* analysed. Scale bar, 200 µm. (*e*) Enlarged image of a fruiting body produced by each strain. Scale bar, 50 µm. (*f*) A graph representing the viability of spores harvested from Ax2, *ampkα^OE^*, *ampkα^−^* and *ampkα^Res^*. The values represent mean ± s.e.; *n* = 4; ***p < 0.001, **p < 0.01 (Student's *t*-test).

To measure the rate of cell proliferation in liquid culture, Ax2, *ampkα^OE^*, *ampkα^−^* and *ampkα^Res^* log phase cells were identically diluted into fresh media at a density of approximately 5×10^5^ cells ml^−1^ and monitored over several days. Ax2 cells reached stationary phase at 12.8 × 10^6^ cells ml^−1^ and cell proliferation of *ampkα^OE^* cells was consistently lower when compared with all other strains and displayed a decline phase after 108 h. *ampkα^OE^* cells proliferated more slowly, reaching stationary phase tardily and at a much lower density of approximately 9.3 × 10^6^ cells ml^−1^. On the other hand, deletion of *ampkα* caused an increase in cell proliferation when compared with the Ax2 cells. The *ampkα^−^* cells reached the stationary phase at a higher cell density of approximately 14.3 × 10^6^ cells ml^−1^ and displayed a decline phase comparatively at an earlier time-point (72 h). *ampkα^Res^* reached stationary phase at approximately 11.2 × 10^6^ cells ml^−1^. The increased proliferation defects shown by *ampkα^−^* cells could be corrected in *ampkα^Res^* up to a certain extent. Doubling times of Ax2, *ampkα^OE^*, *ampkα^−^* and *ampkα^Res^* were 12.1 ± 0.1, 25 ± 0.4, 9.75 ± 0.6 and 15 ± 1.5 h, respectively (figure 2*a*).

In the case of *D. discoideum*, cell proliferation (increase in the number of cells) and cell growth (increase in cell mass or size) are regulated independently. To determine the role of AMPKα in regulating growth, we measured the cell mass of Ax2, *ampkα^OE^*, *ampkα^−^* and *ampkα^Res^* cells. The average cell mass of Ax2 cells was 10.3 ± 0.2 mg and comparable to that observed previously [19]. The average cell mass of *ampkα^OE^*, *ampkα^−^* and *ampkα^Res^* cells were 8.5 ± 0.3, 12.3 ± 0.3 and 11.1 ± 0.2 mg, respectively. Change in the average cell mass of *ampkα^Res^* was insignificant compared with Ax2 cells (figure 2*b*). To determine if AMPKα affects cell size, flow cytometry analyses were performed. Figure 2*c* shows *ampkα^−^* cells to be larger in size, whereas *ampkα^OE^* cells were smaller when compared with Ax2 cells. *ampkα^Res^* cells exhibited similar cell size as Ax2 cells. Taken together, the data suggest that AMPKα regulates both cell proliferation and growth of vegetative cells.

### 2.4. Disruption of *ampkα* gene results in small-sized aggregates, asynchronous development and reduced spore viability

To examine the role of AMPKα in multi-cellular development, we plated cells from the various strains at equal densities (5 × 10^7^ cells ml^−1^) on non-nutrient agar plates and allowed them to develop after synchronization. Ax2 cells completed their development by forming aggregation territories (mounds) by 12 h, slugs at 16 h and finally culminate into fruiting bodies at 24 h. *ampkα^−^* cells developed into smaller aggregates and, subsequently, not all aggregates pursued further development, with only few of them proceeding towards slug formation. Following that, not all slugs proceeded to early culminant at the same time, further added more asynchrony. Thus, the development of *ampkα^−^* cells appeared highly asynchronous, showing various developmental intermediates (aborted mound, slugs, early culminant and culminant) when compared with the Ax2 cells by 24 h of development (figure 2*d*). The fruiting bodies formed by *ampkα^−^* had smaller sori (spore heads) and longer stalks when compared with the Ax2 cells. In fact most of the fruiting bodies formed by *ampkα^−^* cells collapsed, which could be attributed to the longer stalks formed (figure 2*d*). By contrast, the fruiting bodies formed by *ampkα^OE^* cells showed large sori and short stalks when compared with the Ax2 cells (figure 2*e*). As only few fruiting bodies were formed by *ampkα^−^* cells, we checked the viability of the spores. The average viability of Ax2 spores was 84 ± 1.4% [20,21], of *ampkα^OE^* spores was 71 ± 2.8% and of *ampkα^−^* spores was significantly reduced to 45 ± 1.8% (figure 2*f*). The reduced spore viability of *ampkα^−^* was rescued in the *ampkα^Res^*, which displayed average spore viability of 78 ± 4.2% (figure 2*f*).

**Figure 3.**
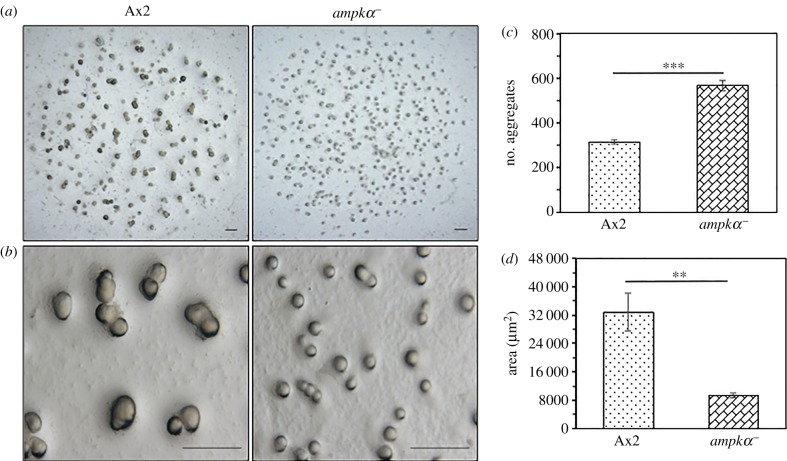
Deletion of *ampkα* leads to small-sized aggregate formation. (*a*) The image shows the complete spot of cells plated for development. Scale bar, 1000 µm. (*b*) Enlarged images of the aggregates formed by Ax2 and *ampkα^−^* cells. Scale bar, 100 µm. (*c*) The graph shows the number of aggregates formed by each strain. (*d*) The graph represents the mound area of Ax2 and *ampkα^−^*. The values represent mean ± s.e.; *n* = 3; ***p < 0.001, **p < 0.01 (Student's *t*-test).

The early developmental defects (small-sized aggregates) shown by *ampkα^−^* were rescued by the exogenous expression of AMPKα, but the late developmental defects (asynchronous development) were still apparent. The *ampkα^−^* cells formed smaller (approx. 3.4-fold decrease) and comparatively more (approx. 1.8-fold increase) aggregation territories than Ax2 cells (figure 3*a–d*).

The above data suggest that an optimum level of AMPKα is necessary for proper development.

### 2.5. *ampkα^−^* cells exhibit changes in secreted factors

**Figure 4.**
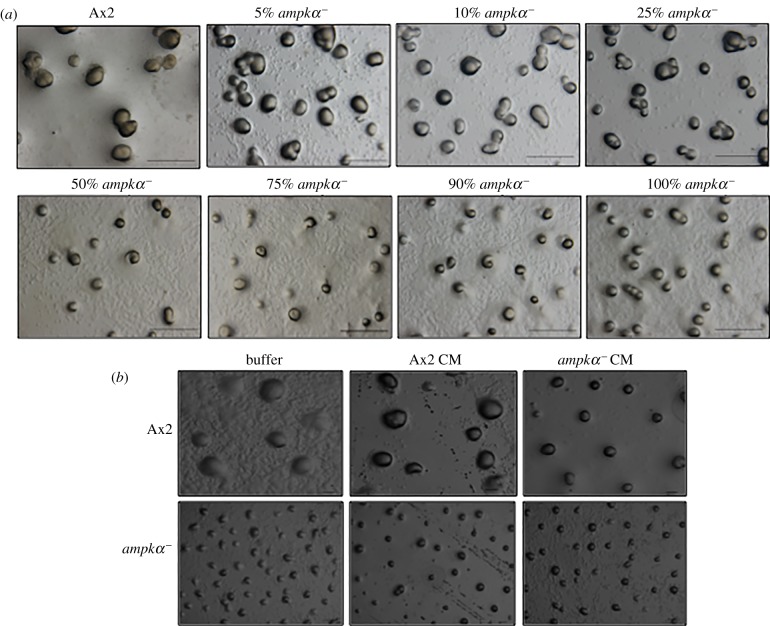
Phenotype of *ampkα^−^* cells is due to changes in the extracellular factors. (*a*) Ax2 and *ampkα^−^* in various ratios (5–90%) were allowed to co-develop to form chimaeric aggregates. (*b*) Development of Ax2 and *ampkα^−^* cells in buffer alone or CM collected from them. CM, conditioned medium; scale bar, 100 µm; *n* = 3.

To investigate the role of secreted factors in the development of *ampkα^−^* phenotype (small-sized aggregates), we performed cell mixing experiments with the Ax2 cells in varying proportions (5–90%) and allowed them to develop. The Ax2 cells in the chimaeras formed were not able to rescue the phenotypic defects caused by *ampkα^−^* cells, suggesting that the small-sized aggregate phenotype was not due to the lack of secreted factor(s). However, with only 5% addition of *ampkα^−^* cells to 95% Ax2 cells, there was a significant increase in aggregate numbers and decrease in aggregate size when compared with Ax2 cells alone (figure 4*a*; electronic supplementary material, figure S3*a,b*). The size of the aggregates in these cell mixtures was indistinguishable from size of those formed by *ampkα^−^* cells alone (figure 4*a*). The size and number of aggregates formed by Ax2 cells were affected by addition of only 5% mutant cells, thus it is possible that mutant cells secrete some factors that regulate group size.

### 2.6. Conditioned medium collected from *ampkα^−^* cells causes the Ax2 aggregates to break up

When cells are starved, they secrete factors that regulate aggregate size. To test the hypothesis that extracellular factors are involved in the phenotype observed by *ampkα^−^* cells, CM experiments were performed. When Ax2 cells were allowed to develop in the presence of CM collected from Ax2 cells, the aggregates formed were similar to the ones formed in the presence of buffer only (figure 4*b*, upper panel). On the other hand, when Ax2 cells were allowed to develop in the presence of the CM collected from *ampkα^−^* cells, the aggregates formed were small-sized and more in number (figure 4*b*, upper panel; electronic supplementary material, figure S3*c,d*). The small-aggregate phenotype was mimicked by the Ax2 cells when developed in the presence of *ampkα^−^* CM. The *ampkα^−^* aggregates were small in size, irrespective of their development in buffer, Ax2 CM or *ampkα^−^* CM (figure 4*b*, lower panel). Therefore, disruption of *ampkα* caused oversecretion of some factors that could regulate the aggregate size.

**Figure 5.**
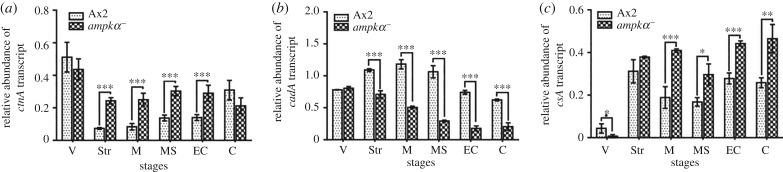
mRNA expression of genes involved in aggregate formation. Relative abundance of the various transcripts involved in aggregation of both Ax2 and *ampkα^−^* cells during development were analysed. (*a*) *ctnA*, (*b*) *cadA* and (*c*) *csA.* V, vegetative; Str, streaming; M, mound; MS, migratory slug; EC, early culminant; C, culminant. The values represent mean ± standard deviation; *n* = 4; ***p < 0.001, **p < 0.01, *p < 0.05 (Student's *t*-test).

As the CM collected from *ampkα^−^* cells allowed the formation of smaller aggregates by the Ax2 cells, we also examined the levels of *countin* mRNA in the *ampkα^−^* cells (figure 5*a*). The expression of *countin* mRNA was significantly increased in *ampkα^−^* cells during development when compared with the Ax2 cells (figure 5*a*). Countin factor (CF) regulates group size by repressing cell–cell-adhesion proteins. The two major cell-adhesion proteins expressed during early development of *Dictyostelium* are Gp24 and Gp80. High expression levels of *gp24* were observed in *countin^−^* cells, whereas *gp80* expression levels were not significantly altered during early development [22]. The mRNA expression of *cadA* (*gp24*) was significantly decreased from streaming onwards in developing *ampkα^−^* cells resulting in decreased cell–cell adhesion, and subsequently resulting in the formation of small-sized aggregates (figure 5*b*). Overexpression of the Gp80 cell-adhesion protein, which causes aggregation streams to break up [23], was significantly increased at the mRNA level from mound onwards in the developing *ampkα^−^* cells, suggesting the possible reason for the break-up of streaming cells to result in small-sized aggregates (figure 5*c*).

### 2.7. Starving *ampkα^−^* cells show low glucose levels

**Figure 6.**
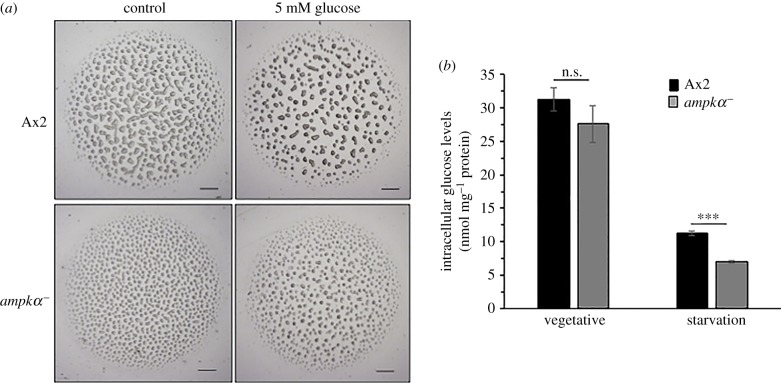
Starving *ampkα^−^* cells exhibit low cytosolic glucose levels. (*a*) Effect of exogenous glucose on aggregates size of Ax2 and *ampkα^−^* cells. (*b*) Cytosolic glucose levels in vegetative and starved Ax2 and *ampkα^−^* cells. Scale bar, 1000 µm. The values represent mean ± standard deviation; *n* = 4; ***p < 0.001 (Student's *t*-test).

CF appears to regulate cytosolic glucose levels and these in turn affect cell adhesion, suggesting that glucose levels are a part of the Countin signal transduction pathway [24]. In other words, glucose affects the group size in *Dictyostelium* and is the downstream target of CF. Adding glucose exogenously negates the effect of high extracellular CF on aggregate size and mimics the effect of depletion of CF. When Ax2 cells were allowed to develop on non-nutrient agar plates containing 5 mM glucose [25], it resulted in the formation of large-sized aggregates (figure 6*a*, upper panel). In our studies, we too found 5 mM of glucose to be the best concentration (data not shown). Our earlier results showed high *countin* expression in the *ampkα^−^* cells; thus, it was possible that the CF could repress the internal glucose levels in these cells. When *ampkα^−^* cells were allowed to develop in the presence of 5 mM glucose, we observed large aggregates, but the size was still comparable to Ax2 (figure 6*a*, lower panel; electronic supplementary material, figure S4*a,b*). Therefore, we measured the cytosolic glucose levels in both the vegetative and starved cells. Cytosolic glucose levels in the vegetative cells of Ax2 (31.26 ± 1.8 nmol mg^−1^ protein) and *ampkα^−^* (27.59 ± 2.8 nmol mg^−1^ protein) were not significantly altered, but the starved *ampkα^−^* cells showed low glucose levels (6.98 ± 0.39 nmol mg^−1^ protein) when compared with the Ax2 cells (11.2 ± 0.18 nmol mg^−1^ protein) (figure 6*b*). Taken together, the data show increased *countin* expression and low glucose levels in the starved *ampkα^−^* cells to be responsible for regulating the aggregate size.

### 2.8. AMPKα regulates spatial cell-type patterning

**Figure 7.**
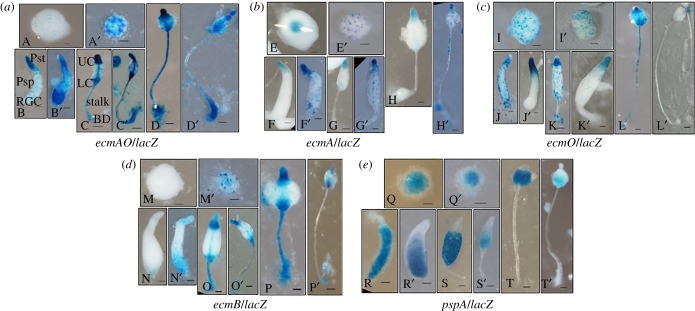
Cell-type-specific expression patterns in Ax2 and *ampkα^−^* developmental structures. Ax2 and *ampkα^−^* cells were transfected with reporter vectors where *lacZ* was expressed under the control of the promoter region from the prestalk-specific genes: (*a*) *ecmAO* [*ecmAO/lacZ*], (*b*) *ecmA* [*ecmA/lacZ*], (*c*) *ecmO* [*ecmO/lacZ*], (*d*) *ecmB* [*ecmB/lacZ*] and (*e*) prespore-specific gene, *pspA* [*pspA/lacZ*]. Pools of transfected cells were developed on nitrocellulose filters. Developmental structures were collected, histochemical X-gal staining was performed and photographs taken. Ax2 (A–T) and *ampkα^−^* (A′–T′) developmental structures; UC, upper cup; LC, lower cup; BD, basal disc; RGC, rear-guard cells; Pst, prestalk; Psp, prespore; scale bar, 50 µm; *n* = 4.

As aberrant fruiting bodies were made by *ampkα^−^* cells, we were interested in elucidating the role of AMPKα in regulating cell-type differentiation (figure 7). We monitored mRNA levels of the cell-type-specific marker genes during development of both Ax2 and *ampkα^−^* cells. Expression levels of the prestalk-specific genes, *ecmA* and *ecmB*, were significantly increased in the *ampkα^−^* cells (electronic supplementary material, figure S5*a,b*) while the expression level of the prespore-specific gene, *pspA* (electronic supplementary material, figure S5*c*), was reduced when compared with the Ax2 cells. We attribute these differences to the formation of fruiting bodies with long stalks and small sori by the *ampkα^−^* cells. We substantiated our results by examining the spatial cell-type patterning using cell-type-specific promoters fused to the *lacZ* reporter in both the Ax2 and *ampkα^−^* cells. We found an increase in the staining region and/or mis-localization of prestalk-specific genes during the development of *ampkα^−^* cells (figure 7*a–d*), while the prespore-specific gene, *pspA*, staining region was reduced (figure 7*e*).

The spatial distributions of prestalk- and prespore-specific genes were compared in the Ax2 and *ampkα^−^* developmental structures (figure 7*a–e*). In the *ampkα^−^* mound, the spatial distribution of *ecmA/lacZ* was aberrant, as it was dispersed all over the mound (figure 7*b*(E′)) rather than only at the tips as in the case of the Ax2 mound (figure 7*b*(E)). The spatial localization of *ecmB/lacZ* was also strikingly aberrant as it was precociously expressed in the *ampkα^−^* loose aggregates (figure 7*d*(M′)) when compared with Ax2 (figure 7*d*(M)) and the staining region of *pspA/lacZ* was decreased in the *ampkα^−^* mound (figure 7*e*(Q′)). The spatial localizations of these genes were also compared in the Ax2 and *ampkα^−^* slugs. In Ax2 slugs, the spatial localization of *ecmAO/lacZ* was found in the anterior region and also distributed throughout the slugs (the anterior-like cells (ALCs)) (figure 7*a*(B)), whereas the *ecmAO/lacZ* staining was expanded in the anterior region of *ampkα^−^* slugs and also more pronounced at the rear-guard region (figure 7*a*(B′)). The *ecmA/lacZ* staining displayed in the anterior region was expanded and aberrant as it was also found in the prespore region of *ampkα^−^* slugs (figure 7*b*(F′)), and the *ecmO/lacZ* spatial localization was expanded in the tip region of *ampkα^−^* migratory slugs (figure 7*c*(J′)) that normally is occupied by pstA cells in Ax2 (figure 7*c*(J)) but was absent in the rear-guard region (figure 7*c*(J′)). The *ecmB/lacZ* expression was found in the anterior region of slugs that was normally occupied by pstA cells and also the staining region was expanded in the rear-guard region (figure 7*d*(N′)), and the *pspA/lacZ* staining was reduced in *ampkα^−^* slugs (figure 7*e*(R′)).

In the *ampkα^−^* early culminant, *ecmA/lacZ* expression was mis-localized as it was also found in the prespore/spore region (figure 7*b*(G′)), *ecmO/lacZ* was absent in the prespore/spore region and the basal disc (figure 7*c*(K′)), *ecmB/lacZ* was absent in the stalk region (figure 7*d*(O′)) and the staining region displayed by *pspA/lacZ* was reduced (figure 7*e*(S′)). In the *ampkα^−^* culminant, *ecmA/lacZ* was also observed in the stalk (figure 7*b*(H′))*, ecmO/lacZ* was absent in the stalk region (figure 7*c*(L′)) and the *pspA/lacZ* staining region was reduced, basically found towards the posterior region of the sorus (figure 7*e*(T′)). In conclusion, we can say that the prestalk/prespore ratio was perturbed and there was a loss of distinct prestalk/prespore boundary in *ampkα^−^* cells. Taken together, our results showed that AMPKα is involved in cell-type patterning and regulates the ratio of prestalk/prespore cells in the multi-cellular structures formed.

### 2.9. Developmental defects displayed by *ampkα^−^* cells are cell autonomous

**Figure 8.**
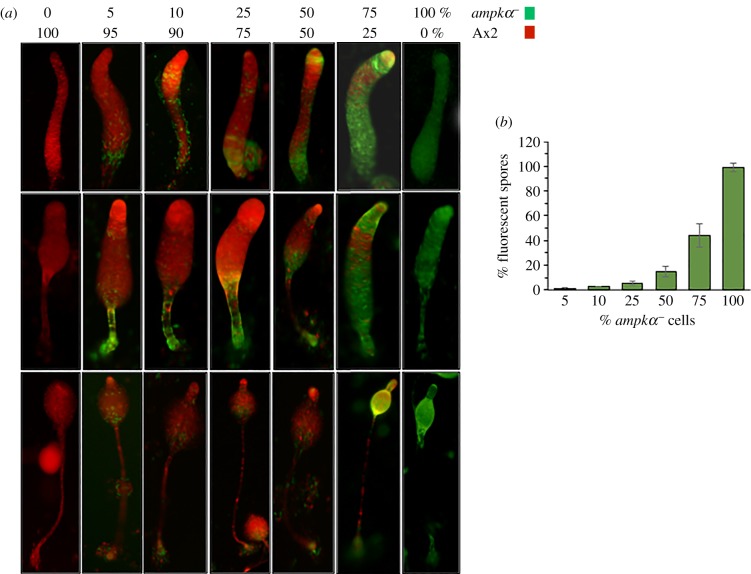
Distribution of Ax2 and *ampkα^−^* cells in chimaeras. (*a*) RFP-tagged Ax2 cells and GFP-tagged *ampkα^−^* cells were mixed in varying proportions and allowed to co-develop. Both DIC and fluorescence images during development (migratory slugs, early culminants and culminants) were captured. GFP-labelled *ampkα^−^* cells populated the prestalk regions (rear-guard and ALCs) of the slugs, whereas RFP-labelled Ax2 cells occupied the prespore regions of the slugs. GFP-labelled *ampkα^−^* populate lower cup and basal disc of early culminant and culminant. (*b*) Percentage (%) spore count of *ampkα^−^* in chimaeric fruiting bodies were scored and represented. Scale bar, 100 µm; *n* = 3.

In the migratory slug, the rear-guard compartment is composed of two prestalk cell types, namely the ALCs and the pstAB-derived cells [26]. The *ampkα^−^* cells in the chimaeras formed with Ax2 cells tend to distribute themselves in the rear-guard region of the slugs formed and in ALCs when mixed in ratios ranging from 5 to 50%, and also show lower tendency to form spores (figure 8). When the percentage of the *ampkα^−^* cells in the chimaeras was increased to 75%, they occupied the prestalk as well as prespore regions but failed to distribute themselves at the boundary of prestalk and prespore regions. This result is consistent with the cell-type-specific marker expression studies, which showed a clear loss of boundary between cell types. Furthermore, this trend also appeared in the chimaeric fruiting bodies formed, where *ampkα^−^* cells distributed themselves largely in the posterior stalk, basal disc (derived from rear-guard cells) and the lower cup of the fruiting bodies (derived from pstO/ALCs) formed (figure 8*a*). Ax2 cells reduced the contribution of *ampkα^−^* cells in chimaeric spore formation (figure 8*b*). Mixing of 5%, 10%, 25%, 50% and 75% of *ampkα^−^* cells with Ax2 cells resulted in chimaeric spore formation in which *ampkα^−^* cells contributed only to 1.22 ± 0.78%, 2.8 ± 0.32%, 5.8 ± 0.32%, 14 ± 4.15% and 44 ± 9.5%, respectively (figure 8*b*). The Ax2 and *ampkα^−^* cells when developed individually could form fruiting bodies on their own. Our data suggest that AMPKα is a critical component responsible for the maintenance of the prestalk/prespore ratio as well as the boundary between prestalk/prespore regions.

## 3. Discussion

During the past several years numerous studies have explored the importance of AMPK in various aspects, but still it needs to be investigated to uncover many unsolved issues. AMPK, a central regulator of metabolism, is a nutrient and energy sensor that is regulated reciprocally upon starvation by either energy or glucose [27–30]. Mammalian AMPK and its homologue present in plants and yeast are known to be activated in response to glucose depletion [31,32] and regulate expression of genes that permit growth on alternative carbon sources [33,34]. Previous findings have shown that AMPK is activated upon decrease in ATP/ADP or ATP/AMP levels [35]. The change in ATP/ADP level could be the consequence of various factors like glucose depletion, hypoxia, reactive oxygen species, etc. [29,36]. In *Dictyostelium*, multi-cellular development programme is initiated upon nutrient starvation and cells undergo various metabolic changes to survive and further differentiate. The major energy sources for starving cells result from the cellular degradation of RNA and protein, but glycolysis and gluconeogenesis contribute minimally [37].

We have characterized AMPKα from *D. discoideum* and show its involvement in the regulation of aggregate size and cell-type patterning. *Dictyostelium* AMPKα, like other AMPK homologues, is activated upon starvation (electronic supplementary material, figure S6), suggesting its role in nutrient sensing. The mRNA is expressed at all stages of development and is localized in the dying cell population, suggesting it to be involved in stalk cell differentiation and also to play a role in cell differentiation.

### 3.1. AMPKα regulates aggregate size

**Figure 9.**
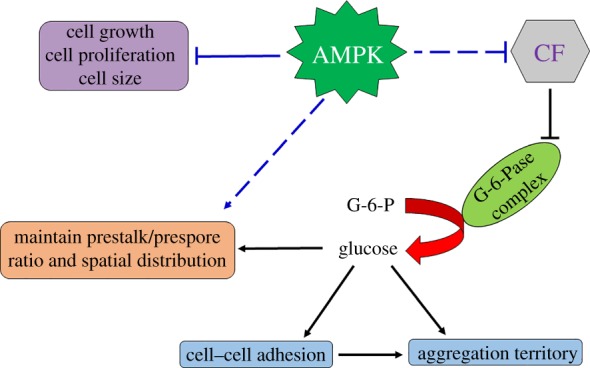
Model illustrates the role of AMPKα in growth and development. AMPKα inhibits cell growth, proliferation and size. It inhibits CF that represses the conversion of glucose from glucose-6-phosphate to control cellular events such as cell–cell adhesion, aggregation territory size, prestalk/prespore ratio and spatial cell-type distribution. AMPKα also helps in maintaining the prestalk/prespore ratio and spatial distribution. In the above model, solid lines indicate known information, while dotted lines display proposed hypothesis from this study.

One of the most interesting aspects of development is how a large number of cells organize themselves into structures of specific sizes and shapes. In this study with *D. discoideum*, we have identified AMPKα in regulation of aggregate size. We show the mutant *ampkα^−^* cells to have a defect in aggregate size determination, which causes the formation of a high density of aggregates containing small numbers of cells. We found (data not shown) that when plated at higher density for development, they formed smaller aggregates, showing there is a defect in the size-determination mechanism rather than an inability to form aggregates. In fact, the defect was not limited only to the aggregate size-determination mechanism, as the mutants also showed defects in cell proliferation, development and differentiation of prespore and prestalk cells. It is known that CF (a 450 kDa complex of five proteins), a component of CM, is secreted in moderate amounts by the Ax2 cells, and regulates the number of cells present within the group and ultimately controls the size of the aggregates. Disruption of the *countin* gene does not allow the aggregation stream to break up, thus resulting in the formation of large aggregates and subsequently larger fruiting bodies, while oversecretion of Countin protein causes the aggregation streams to break up resulting in smaller aggregates and ultimately smaller fruiting bodies [11,38]. The small aggregates are due to an increased amount of secreted factor by the *ampkα^−^* cells, as *ampkα^−^* phenotype could be imitated by developing the Ax2 cells in the presence of the CM collected from the mutant cells, but it also could not be rescued by starving *ampkα^−^* cells in the presence of Ax2 CM. The ability of 5% *ampkα^−^* cells in a population of 95% Ax2 cells to cause the entire population to form small aggregates suggests that the mutant phenotype is not due to the lack of secreted factor. As AMPK is a serine/threonine kinase and regulates the expression of a variety of proteins, it possibly could regulate the group size by regulating *countin* expression which involves levels of intracellular glucose and also represses the cell-adhesion proteins. The *ampkα^−^* cells exhibit increased *countin* expression, which reduces the glucose levels and exhibits aberrant cell-adhesion expression to maintain the group size. From our studies, we conclude that in wild-type cells, AMPK represses Countin and subsequently the glucose levels and cell-adhesion proteins to regulate the aggregate size (figure 9). The *ampkα^−^* cells developed into comparatively larger aggregates in the presence of exogenous glucose, but the size still remained comparable to wild-type. Therefore, we could say that decreased glucose levels were not the only cause of small-sized aggregates, but there may also exist some other factors that directly or indirectly regulate aggregate size.

An enigmatic aspect of AMPKα is that disruption caused increase in cell growth and proliferation; thus, one would think that the absence of it is advantageous to the cell. A vital aspect of *Dictyostelium* development is formation of the fruiting body for maintaining its generation. The spores thus produced are a measure of one's fitness and germinate upon favourable conditions. The *ampkα^−^* and *ampkα^OE^* cells both formed aberrant fruiting bodies and the spores displayed reduced viability when compared with Ax2. Therefore, abnormally low or high levels of AMPKα reduce the ability of spores to germinate and an optimum level is necessary for efficient spore formation. The function of the fruiting body is to lift the spore mass as high off the ground as possible, for optimal spore dispersal. If the fruiting body is too large, it would collapse and the spores on the ground will not get dispersed. Therefore, there is a limit on the number of cells in the spore mass as well as in the stalk of a fruiting body. As the mutants do complete their development and form fruiting bodies, we believe that all other components required for development are below the acceptable range.

### 3.2. AMPKα helps maintain prestalk/prespore ratio and spatial cell-type patterning

In the case of *Dictyostelium,* cell differentiation is initiated when myxamoebae stop growing and dividing. During development, cells terminally differentiate into two main cell types, the stalk and the spore cells, whose precursors are prestalk and prespore, respectively, and are observed at earlier developmental stages and distinguished by marker genes. The ratio of prestalk and prespore cells remains constant regardless of the size of the slug, and any alteration in this would lead to aberrant morphogenesis and differentiation [15,39,40]. Previous reports with *Dictyostelium* showed cells grown in the presence of glucose (G^+^ cells) had increased spore-to-stalk as well as prespore-to-prestalk ratio when compared with the cells grown in glucose-depleted conditions [41,42]. The study was also supported by other groups who showed that cells when grown in the absence of glucose (G-) developed fruiting bodies with long stalks and small sori when compared with the cells grown in glucose-rich conditions, attributed to the differences in spore-to-stalk ratio [42,43]. Cell-type-specific marker expression studies in *ampkα^−^* cells display an increase in prestalk-specific gene expression and concomitant reduction in prespore-specific gene expression, and also the spatial localization of these genes was altered. In our study, we found that starved *ampkα^−^* cells had low glucose levels and developed fruiting bodies with long stalks and small sori. Therefore, reduced glucose levels could be one of the causes for this inequitable fruiting body formation. Mixing of cell types in the *ampkα^−^* mutant suggest its role in maintaining the boundary between them. Thus, AMPKα assists in proportioning and patterning of prestalk/prespore cells.

Glucose has also been shown to play a role in the cell sorting behaviour of *Dictyostelium* cells. The differential preferences of cells in development and differentiation have been observed in various organisms using chimaera study. Chimaeras are mixture of cells from two or more genetically different backgrounds. Several chimaeras in *Dictyostelium* show unequal apportionment of the genetically different cells into spore and stalk cells [21,44]; the one that gets a higher proportion of spores is called cheater and the other is called loser [45]. Khare & Shaulsky [46] have led this concept of multi-cellular bodies using competition to place their best cells into justified functions, so the question arises, do the mutant cells contribute equally in spore formation of chimaeras? *ampkα^−^* cells form few fruiting bodies from a given number of cells when compared with the wild-type cells in pure populations; this result is sufficient to explain the competitive failure of mutant cells in contributing to spore formation in chimaeras. There are various reports that explain the role of glucose in the sorting behaviour of *Dictyostelium* cells during differentiation. In chimaeras, G^+^ cells preferentially become prespore cells of the slug and G*^−^* cells sort out to become prestalk cells [47–49]. Our study provides insights about the differential preferences of *ampkα^−^* cells that exhibit low glucose levels in the cell sorting behaviour*.* The *ampkα^−^* cells participate mainly in prestalk-derived structures and have less proclivity to occupy the prespore/spore region within chimaeras in the presence of Ax2 cells. Previous studies showed deletion of *ampkα* resulted in embryonic lethality and, therefore, its direct impact on cellular differentiation and lineage choice is still unknown [50]. Young *et al*. [51] showed direct impact of AMPKα in cell fate determination during differentiation. Further studies are still required to understand the direct impact of AMPKα on cell lineage tracing and differentiation in *Dictyostelium*.

## 4. Conclusion

Our results demonstrate that AMPKα regulates aggregate size in *Dictyostelium.* The fruiting bodies formed by *ampkα^−^* cells result from the differences in prestalk/prespore ratio. The results also suggest a role in cell-type differentiation and spatial patterning.

## 5. Material and methods

### 5.1. Growth and development of *Dictyostelium discoideum*

The *Dictyostelium* cells were grown and developed as described [52]. The logarithmic phase cultures (2.5–5 × 10^6^ cells ml^−1^) were identically diluted into fresh media at a density of approximately 5 × 10^5^ cells ml^−1^ and monitored over 6 days for measuring cell proliferation.

For development, exponentially growing cells were harvested, washed in 1×KK_2_ buffer and spotted at a density of 5 × 10^7^ cells ml^−1^ on non-nutrient agar plates. The plated cells were incubated at 4°C for 4–6 h for synchronization of the development, followed by incubation at 22°C. Analyses of mound size were performed using NIS Elements AR v. 4.0.

### 5.2. RNA detection by RT-PCR and *in situ* hybridization analyses

RNA was isolated as described [52] from growing amoebae and at various developmental stages. RT-PCR reactions were performed using gene-specific primer pairs (electronic supplementary material, table S1). *rnlA* (*ig7*) was used as an internal control.

The *in situ* hybridization studies were performed as described [52]. The probe was obtained by cloning a 744 bp PCR amplified region using the gene-specific primer pairs into XhoI/XbaI site of commercially available pBluescriptII phagemid (pBSIISK+) vector (electronic supplementary material, figure S7). It was followed by *in vitro* transcription into RNA, sense probe with T7 polymerase (template obtained after construct digestion with XbaI) and antisense probe with T3 RNA polymerase (template obtained after construct digestion with XhoI) were obtained. The probe was labelled with a DIG RNA labelling kit (Roche Diagnostics) and *in situ* hybridization was performed. A sense probe was used as a control.

The list of primer pairs for other genes used in this study is given in the electronic supplementary material, table S1.

### 5.3. Preparation of construct and strains

#### 5.3.1. *ampkα* overexpressing strain

A full-length *ampkα* gene was PCR amplified (electronic supplementary material, figure S1) from genomic DNA using the gene-specific primer pairs (electronic supplementary material, table S1) and cloned into *act15/Acg-Eyfp* vector (electronic supplementary material, figure S1) that contains the *actin 15* promoter, enhanced yellow fluorescent protein (Eyfp) at the C-terminus, a G418 resistance marker for selection in *Dictyostelium* and an ampicillin resistance marker for selection in bacteria.

#### 5.3.2. *ampkα^−^* strain

The 5′ and 3′ targeting regions were PCR amplified (electronic supplementary material, figure S2) by using the gene-specific primer pairs (electronic supplementary material, table S1) and introduced into the flanking side of the Blasticidin (Bsr) cassette. After restriction digestion, linearized knockout construct was introduced into Ax2 cells by electroporation and the transformed cells were selected at 10 µg ml^−1^ Blasticidin-S (Invitrogen). Genomic DNA was extracted as described [53] and targeted gene disruptions were identified by several PCR reactions (electronic supplementary material, figure S2*e*) using primers (electronic supplementary material, table S2), and the absence of protein was further confirmed by western hybridization (electronic supplementary material, figure S2*f*).

#### 5.3.3. *ampkα* rescue strain

The rescue strain was made by expressing an AMPKα–Eyfp fusion construct into *ampkα^−^* cells.

### 5.4. Cell dry weight

Nearly 5 × 10^7^ log phase cells were washed and vacuum dried at 55°C. After 1 h, the weight of dry pellets was scored.

### 5.5. Flow cytometry

To determine cell size, a BD FACS Calibur flow cytometer with Cell Quest software was used. About 1 × 10^7^ log phase cells were harvested, washed in 1×KK_2_ buffer and re-suspended in 1.5 ml buffer (0.9% NaCl, 2% sucrose, 5 mM EDTA in KK_2_ buffer). Cells were fixed by adding 75% chilled ethanol dropwise and incubated for 30 min at 22°C and stored at 4°C. Immediately before analysis, 1 × 10^6^ cells were washed, re-suspended in KK_2_ buffer, and incubated at 37°C for 30 min in 10 µg ml^−1^ RNase A (Sigma-Aldrich, USA) followed by incubation at room temperature in 50 µg ml^−1^ propidium iodide (Sigma-Aldrich).

### 5.6. Conditioned medium experiments

CM was prepared as described previously [54]. Briefly, log phase cells of Ax2 and *ampkα^−^* were starved and re-suspended at a density of 1 × 10^7^ cells ml^−1^ and kept under shaken conditions for 20 h, at 22°C. Cells were pelleted down and the supernatant was further clarified by centrifugation. The clarified supernatant thus prepared or CM was used immediately. To check the effect of CM on aggregate size, Ax2 and *ampkα^−^* cells were starved on dialysis membranes, which were placed on Whatman filters (two to three layers) soaked in Ax2 CM or *ampkα^−^* CM. Cells starved on Whatman filters (two to three layers) soaked in 1×KK_2_ buffer were used as a control.

### 5.7. Spore viability assays

Spore viability assay was followed as described [21]. Spores from mature fruiting bodies were harvested in spore buffer (40 mM KH_2_PO_4_, 20 mM KCl, 2.5 mM MgCl_2_), washed twice by centrifugation at room temperature and counted in a haemocytometer. Aliquots of 100 spores were mixed with a suspension of bacteria (*Klebsiella aerogenes*) and grown for 5 days. The per cent viability of spores was measured by counting the number of clear plaques formed on the bacterial lawns divided by total spores plated followed by multiplication with 100. Three independent experiments in triplicate were performed.

### 5.8. Glucose assay

Glucose assay was performed as described [25] with minor modifications. Log phase cells were harvested and re-suspended at a density of 8 × 10^6^ cells ml^−1^ and kept under shaken conditions for 6 h at 22°C. Cells were harvested and lysed by the freeze–thaw method. Glucose assay was performed as per instructions given by the manufacturer (GAHK20; Sigma-Aldrich). Protein levels were measured using Bradford reagent (BIO-RAD).

### 5.9. Development of chimaeric mixtures

Ax2 and *ampkα^−^* cells were transfected with pTX-RFP and pTX-GFP, respectively, and selected at 40 µg ml^−1^ of G418. GFP- and RFP-marked cells were mixed in varying percentages (5–75%) and developed on non-nutrient agar plates. Both DIC and fluorescent images were captured on a Nikon SMZ-1500 microscope. Individual spore heads were picked on a glass-slide and photographed, both under brightfield and under fluorescence using a Nikon eclipse 80i fluorescence microscope. Red and green fluorescent spores were counted from the photographs. A minimum of 10–15 fruiting bodies developed from each mixture per individual experiment was counted.

### 5.10. β-Galactosidase staining

β-Galactosidase staining was performed as described [52]. Images were captured using a Nikon AZ100 microscope.

### 5.11. Statistical analysis

The statistical analyses were performed (mean standard deviation and standard error) and values were plotted in graph using Microsoft Excel-2013 and GraphPad Prism. *p*-Values of less than 0.05 were considered as significant.

## Supplementary Material

Electronic supplementary file

## Data accessibility

The datasets supporting this article have been uploaded as part of the electronic supplementary material.
