# Supplementary material for: Dictyostelium AMPKα regulates aggregate size and cell-type patterning
Source: Open Biol. 2017 Jul 12;7(7):170055. doi: 10.1098/rsob.170055 (PMC5541345; doi:10.1098/rsob.170055)
Supplement: Electronic supplementary file [file rsob170055supp1.pdf]

***Dictyostelium* AMPKα regulates aggregate-size and cell-type patterning**

Ranjana Maurya, Rakesh Kumar and Shweta Saran*

School of Life Sciences, Jawaharlal Nehru University, New Delhi-110067

*Corresponding author: Shweta Saran

School of Life Sciences, Jawaharlal Nehru University, New Delhi, India-110067

Email: ssaran@mail.jnu.ac.in; shweta_saran@hotmail.com

RM: ranjanamauryasls@gmail.com

RK: rakeshkapoor.jnu@gmail.com

Telephone No.: (O) +91 11 26704157

(M) 09871382398

All the authors approved the final manuscript before submission.

There is no conflict of interest.

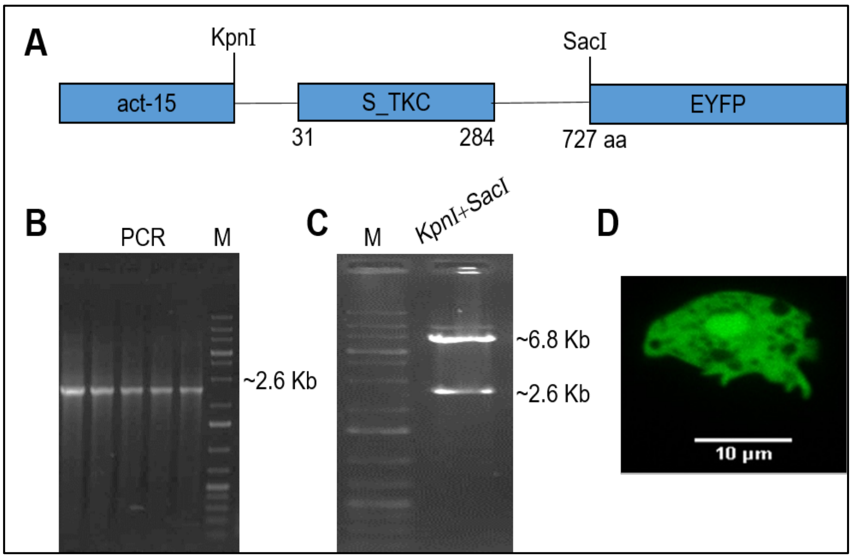

Figure S1. Construction of the overexpressing strain *(ampkα^{OE})*. (A) Diagrammatic representation of *ampkα* overexpression construct. (B) PCR amplification of the *ampkα* gene using gene-specific primers from the genomic DNA. (C) Confirmation of clone yielded 6.8 Kb vector backbone and a ~2.6 Kb insert. (D) Transformed cells expressing AMPKα–Eyfp fusion protein. [M- DNA Marker].

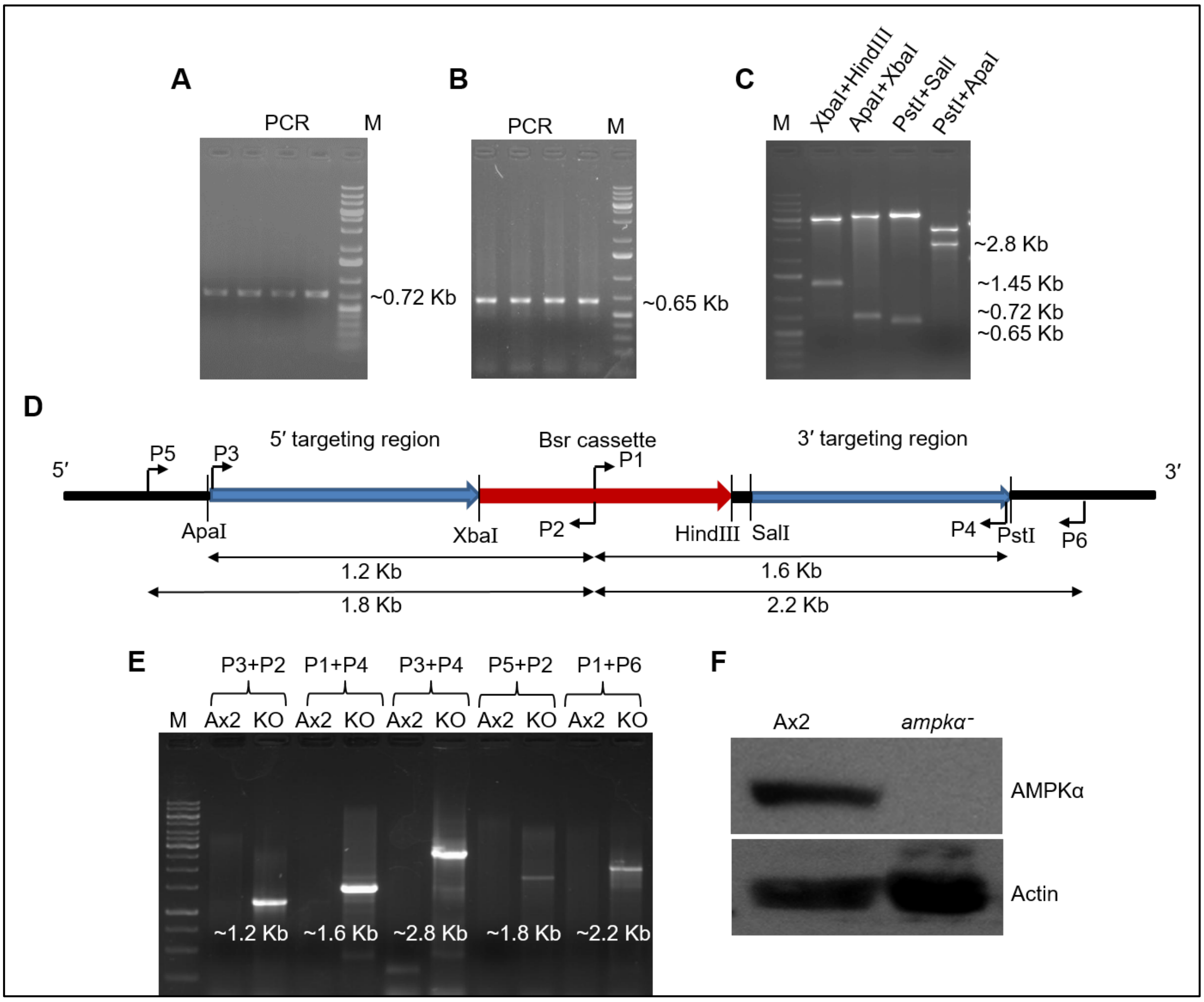

Figure S2. Creation and confirmation of *ampkα* knockout construct and strain. (A) PCR amplification of the 5′ targeting region was approximately 0.7 Kb. (B) PCR amplification of the 3′ targeting region was approximately 0.65 Kb. (C) Confirmation of 5′ and 3′ targeting region and bsr cassette via restriction digestions. (D) Diagrammatic representation of *ampkα* knockout construct obtained. The primer positions and expected amplicons sizes are marked. (E) Confirmation of *ampkα* knockout strain by PCR from Ax2 and *ampkα*$^-$ genomic DNA using various primer combinations. (F) Whole-cell extracts were prepared from Ax2 and *ampkα*$^-$ vegetative cells and AMPKα protein expression was measured by western blotting (anti-AMPKα antibody; CST and anti-Actin antibody; CST). [M- DNA Marker; P1, P2, P3, P4, P5 and P6 are different primers used for knockout screening.]

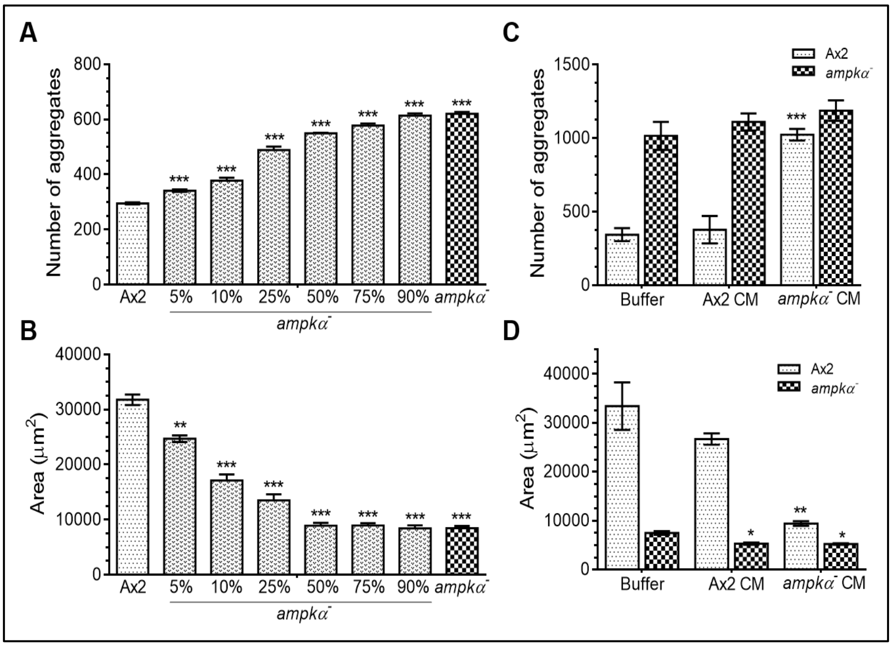

Figure S3. Comparison of aggregates number and size. (A) and (B) Graph shows the number and size of aggregates formed in various chimaeric mixtures (5-90% *ampkα*⁻ cells). *ampkα*⁻ cells cause the Ax2 aggregates to break up into smaller one. (C) and (D) Graph representing the aggregates number and size formed by Ax2 and *ampkα*⁻ cells in buffer, Ax2 and *ampkα*⁻ conditioned medium. Ax2 cells form smaller aggregates when developed in *ampkα*⁻ CM, whereas *ampkα*⁻ cells were small-sized in both Ax2 and *ampkα*⁻ CM. Values were compared and significance was plotted against control. [The values represent mean ± standard deviation; n=4; ***P < 0.001, **P <0.01, *P < 0.05 (Student's t-test)].

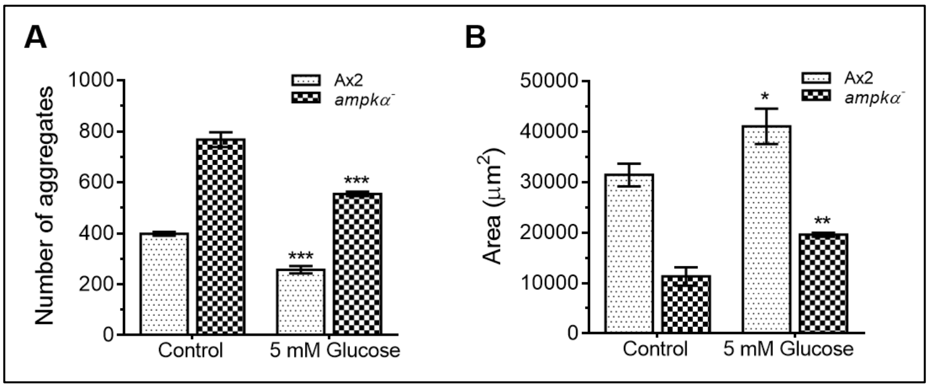

Figure S4. Comparison of aggregate numbers and sizes formed in the presence of exogenous glucose. (A) and (B) Graph shows the number and size of aggregates formed by Ax2 and *ampkα*⁻ cells in presence of 5 mM glucose. Values were compared and significance was plotted against control. [The values represent mean ± standard deviation; n=4; ***P < 0.001, **P <0.01, *P < 0.05 (Student's t-test)].

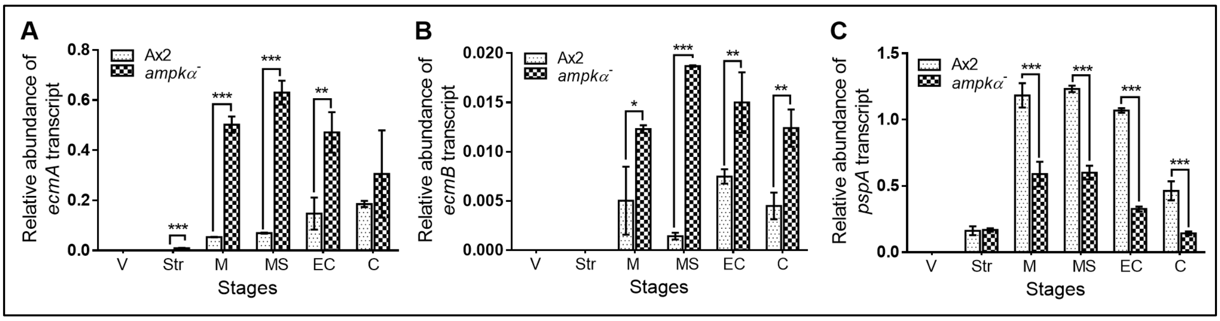

Figure S5. Analyses of cell-type specific marker gene expression during development. Relative abundance of the various transcripts in both, Ax2 and *ampkα*⁻ cells during development were analysed. (A) *ecmA*, (B) *ecmB* and (C) *pspA*. [V- Vegetative, Str- Streaming, M- Mound, MS- Migratory Slug, EC- Early Culminant, C- Culminant. The values represent mean ± standard deviation; n=4; ***P < 0.001, **P <0.01,*P < 0.05 (Student's t-test)].

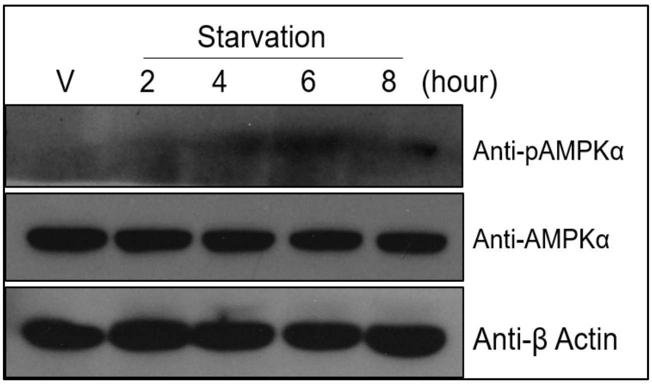

Figure S6. Western blots showing AMPKα activation during starvation. Ax2 cells were starved in 1xKK_2 buffer and samples were collected every 2 hours till 8 hours. Cell lysates were prepared and protein expression was measured by western blotting using phosphorylated AMPKα (pThr172) and AMPKα antibody. β- Actin was used as a loading control. [V- Vegetative; all antibodies were purchased from Cell SignallingTechnology].

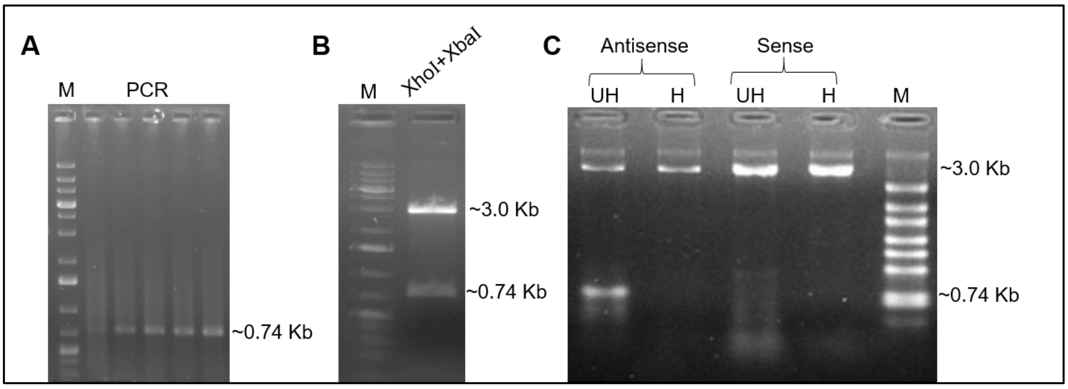

Figure S7. Cloning for *in-situ* hybridisation studies. (A) PCR amplification of ~0.74 Kb genomic region for the preparation of probe. (B) Confirmation of *in-situ* clone yielded 3.0 Kb vector backbone and a ~0.74 Kb insert. (C) The unhydrolysed and hydrolysed product of antisense and sense probes. [M- DNA Marker].

Table S1: List of oligonucleotides used in this study. Sequence underlined marks the restriction enzyme site.

Gene name	Primer	Primer sequence (5′------- 3′)	Genomic position
in-situ	Forward	CATCCTCGAGCCAAGCAATAGCAATAGCATCAG	1514-1536
	Reverse	GCTCTAGAATGTGGTTGGGTTTGAATATCTG	2235-2257
ampkα RT	Forward	GGAAATAGTAATGGTAGCAACAGC	1859-1882
	Reverse	CTTTCCATTAACCATTCTACATCTT	2495-2519
ampkα^{OE}	Forward	CATCGGTACCAGTTCATATCAACAAAATCCCATT	4-27
	Reverse	CTACGAGCTCCAATTCCTCCAACATTAAACTACAAA	2617-2642
ampkα 5′ targeting region	Forward	CGATGGGCCCTTTGGTGGTCTATATGGCAGTGGA	34-57
	Reverse	GACTTCTAGACAGAGATAACTTCAGGTGCAGCGTAAT	731-757
ampkα 3′ targeting region	Forward	CTCAGTCGACGTAATGGTAGCAACAGCAATAACAA	1866-1890
	Reverse	GTGACTGCAGGGCTTTCCATTAACCATTCTACATC	2497-2521
rnlA RT	Forward	TGAATTGAAGTCTGAGTAAACGG	1795-1817
	Reverse	TAGATAGGGACCAAACTGTCTCAC	3065-3042
ctnA RT	Forward	ATTTTAGCTTTATTCCTTGTCAAC	22-45
	Reverse	GTGTAAGCAATTGAGAGGGTGAAT	468-491
cadA RT	Forward	TCTGTTGATGCAAATAAAGTAAAA	4-28
	Reverse	ATAGTCATATGGTGTATGTGTTTG	583-559
csA RT	Forward	GTGAACGACTCTATTAACTCTGCT	406-430
	Reverse	AGTTGGAGTGTCTGGAATTGTATA	1374-1350
ecmA RT	Forward	GATGATGGAAATAGATGGTCAACA	220-243
	Reverse	GTACATTGGTTATTATCATCGACA	1236-1213
ecmB RT	Forward	GTGGTGTTACTCATACTCCAATTCGTT	2611-2637
	Reverse	CATTGGAACATGAATACATTTACCACC	3366-3340
pspA RT	Forward	GATAGGATCCCCAGTTTGTGCTTCAGTAGATGTC	94-117
	Reverse	ACTTCTCGAGGTTGTTGTTGATGTTTGGGATGG	434-415

Table S2: List of oligonucleotides used for *ampka* knockout screening.

Gene name	Primer	Primer sequence (5′------- 3′)
Primers for *ampkα* knockout screening	P1	TTTGTCCATTCGAAACTGCA
	P2	TGCAGTTTCGAATGGACAAA
	P3	CGATGGGCCCTTTGGTGGTCTATATGGCAGTGGA
	P4	GTGACTGCAGGGCTTTCCATTAACCATTCTACATC
	P5	TCCAGCCCTATATATATCCAC
	P6	TAAGTATTGATAAAAAGAGCACTC
